# A Comparison of Arch Length, Intercanine Width, Intermolar Width, and Interalveolar Width in Patients With Angle’s Class II Division I Malocclusion With Shallow and Deep Palates

**DOI:** 10.7759/cureus.71054

**Published:** 2024-10-08

**Authors:** Syeda Ruiya, Rajesh RNG, Anadha N Gujar, Rony T Kondody, Nandaprasad Shetty, Swati Vishwakarma, Roopa K Murthy, Anushree MK

**Affiliations:** 1 Orthodontics and Dentofacial Orthopaedics, Sri Rajiv Gandhi College of Dental Sciences and Hospital, Bengaluru, IND; 2 Oral Pathology, Sri Rajiv Gandhi College of Dental Sciences and Hospital, Bengaluru, IND

**Keywords:** angle’s class ii division 1, arch length, interalveolar width, intercanine width, intermolar width, intra-arch dimensions, palatal depth, skeletal class ii malocclusion

## Abstract

Background and objective

Palatal depth is significant as it impacts the intra-arch dimensions in patients with Angle’s Class II Division 1 malocclusion. Hence, understanding this relationship is crucial for developing targeted orthodontic treatment plans, to optimize dental alignment and overall facial aesthetics. This study aimed to compare the arch length, intercanine width, intermolar width, and interalveolar width in patients with Class II Division 1 malocclusion with deep palates and those with shallow palates.

Methods

The study involved 88 patients aged 18-30 years, categorized into two groups based on palatal depth: Group A (deep palates) and Group B (shallow palates). Measurements were taken for arch length, intercanine width, intermolar width, and interalveolar width. An independent samples t-test was used to compare these measurements between the groups, with statistical significance set at p<0.05.

Results

The analysis revealed significant differences in intermolar and interalveolar widths between the two groups. Group B, with shallow palates, showed larger intermolar and interalveolar widths compared to Group A, with deep palates (p = 0.001 and p = 0.017, respectively). However, no significant differences were observed in arch length and intercanine width between the groups.

Conclusions

Our findings suggest that palatal depth is significantly associated with certain intra-arch transverse dimensions, particularly intermolar and interalveolar widths. These differences underline the importance of considering palatal morphology in the orthodontic treatment of patients with Class II Division 1 malocclusion.

## Introduction

The growth of the craniofacial structures is an ongoing process with significant changes occurring during the late mixed dentition period. A balanced facial form and function arise from the harmonious integration of the various elements of this complex, which grow and develop sequentially and predictably, though there is considerable variation in the amount and timing of growth [1]. Craniofacial development and malocclusion are influenced by several factors, such as tooth size, arch size and shape, the number and arrangement of teeth, jaw size and relationships, and associated soft tissues like lips, cheeks, and tongue. Research and clinical observations have established that heredity and environmental factors significantly influence craniofacial development and play a key role in the emergence of malocclusions [2].

Among various malocclusions, Class II malocclusion is a common condition frequently encountered in routine practice by orthodontists; it can be classified as either skeletal or dental, though it often involves a combination of both factors [3]. Skeletal Class II malocclusion may be associated with a retrognathic mandible, a prognathic maxilla, or both whereas dental Class II malocclusion is characterized by a distalized position of the mandibular molars and canines relative to the maxillary molars and canines [4,5]. Class II Division 1 malocclusion is frequently characterized by the upper incisors inclined labially, resulting in an increased overjet, relatively narrow maxillary arch with subsequent reduction in the transverse dimension, and the vertical overlap of the incisors which can vary from a deep overbite to an open bite [6,7].

The structure of the palate is a crucial aspect in Class II malocclusion, as palatal morphology significantly influences an individual's skeletal and facial patterns. Research indicates a correlation between palatal depth and Class II malocclusion. Buschang et al. [8] observed that individuals with Class II Division 1 malocclusion exhibit greater palatal height and a narrower maxillary dental arch compared to those with Class II Division 2 malocclusion. Similarly, Singh [9] reported that subjects with Class II Division 1 malocclusion often have a deep palatal vault. However, palatal morphology varies considerably in its vertical dimensions. Nahidh et al. [10] observed that palatal height was lower in Class II Division 1 malocclusion, with no significant differences when compared to other malocclusions considered in the study. This finding aligns with the research by Paoloni et al. [11], which evaluated morphometric covariance between palatal shape and skeletal pattern in Class II Division 1 subjects and revealed the presence of both deep and shallow palates associated with malocclusion. Previous studies showed conflicting findings regarding the palatal depth of Class II Division 1 cases though most cases were closely associated with a narrow and high palate.

Moreover, studies evaluating arch dimensions have shown a general trend of reduced maxillary arch width in individuals with Class II Division 1 malocclusion. Research by Varrela [12] and Rejman et al. [13] reported significantly smaller intercanine and intermolar widths compared to those with normal occlusion, especially in cases with crowding. However, other studies, such as that by Frohlich [14], who found no significant differences in intra-arch widths between Class II malocclusion and normal occlusion, and Shamaa [15], who observed significantly smaller maxillary intermolar widths in Class II Division 1 patients, present conflicting results. Sayin and Turkkahraman [16] further supported the finding of reduced maxillary intermolar width in Class II Division 1 patients compared to normal occlusion.

While the impact of arch dimensions and palatal depth on Class II Division 1 malocclusion has been partially explored, the correlation between palatal depth and transverse arch dimensions in these patients remains inadequately studied. Previous studies have focused on either palatal morphology or arch dimensions in isolation, but few have comprehensively assessed the interplay between these factors in determining the severity and characteristics of Class II Division 1 malocclusion. Hence, this study aimed to compare the arch length, intercanine width, intermolar width, and interalveolar width in patients with Class II Division 1 malocclusion with deep palates and those with shallow palates.

## Materials and methods

The study was conducted in the Department of Orthodontics and Dentofacial Orthopedics at Sri Rajiv Gandhi College of Dental Sciences and Hospital in Bengaluru, Karnataka. Ethical clearance was obtained from the institution's ethics committee (No. SRGCDS/2023/235). Considering the standard deviation of 4.94 and 3.21 and mean difference of 2.48 from the parent article, the calculated effect size amounted to 0.6085, with 5% alpha error and 80% power; the calculated sample size came up to 44 per group.

In this study, lateral cephalograms of patients were taken based on the following inclusion and exclusion criteria. The inclusion criteria were as follows: patients in the age group of 18-30 years, Skeletal Class II (ANB >2°), Angle’s Class II Division I malocclusion, with lateral cephalograms taken in the natural head position. The exclusion criteria were as follows: patients with a previous history of orthodontic treatment, subjects exhibiting functional shifts and gross facial asymmetries, a history of habits that may affect the dentofacial region, patients with grossly carious teeth and multiple missing teeth, history of trauma, and patients with syndromes, systemic conditions, or long-term medication use.

Methodology

In this study, the subjects with ANB angle greater than 2^o^ were selected and were categorized into two groups. Group A consisted of 44 individuals with Angle’s Class II Division 1 malocclusion having a deep palate, whereas Group B consisted of 44 individuals with the same malocclusion but a shallow palate. Qualitative assessment of palatal depth typically categorizes it as high, moderate, or shallow. The following formula was employed to determine the palatal height index using study models of the participants, which evaluated the height of the palate in the molar region [17].

Palatal height index = (height of the palate x 100)/width of the palate

The depth of the palate was categorized into three types based on this formula and the study conducted by Maria CM [17]: (a) low palate with values ≤27.9%, (b) medium palate with values ranging between 28.0 and 39.9%, (c) high palate with values greater than 40.0% [17].

**Figure 1:**
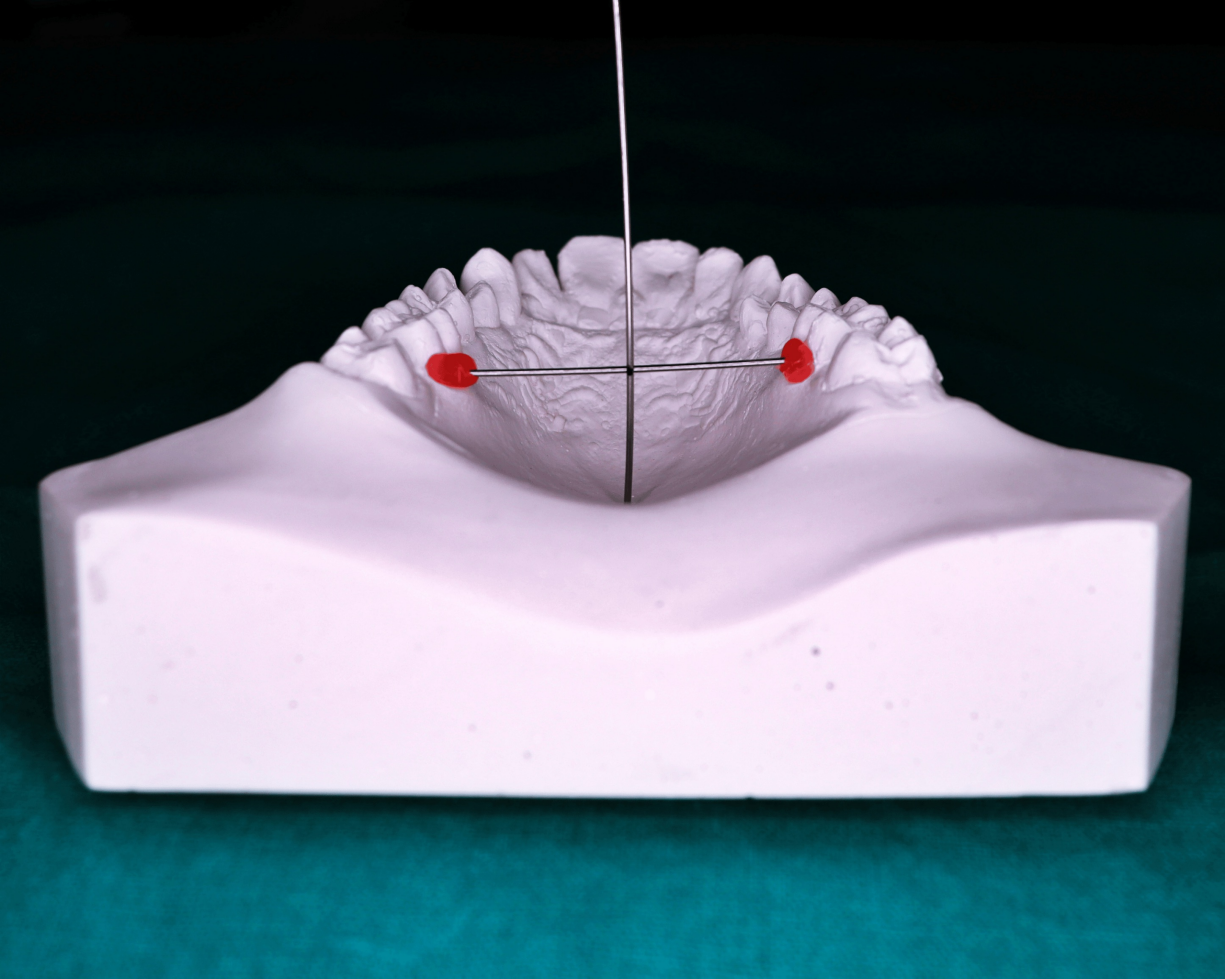
Measurement of palatal height index

To measure palatal height, a stainless steel wire that was cut to the appropriate transverse breadth of the palate was waxed and fastened to the gingival sulcus edges of the first molars. Using a second stainless steel wire, the height was determined by deducting 0.05 mm from the perpendicular distance between the palate and the stainless steel wire (the diameter of the steel wire secured with red wax for palatal width) (Figure 1).

**Figure 2:**
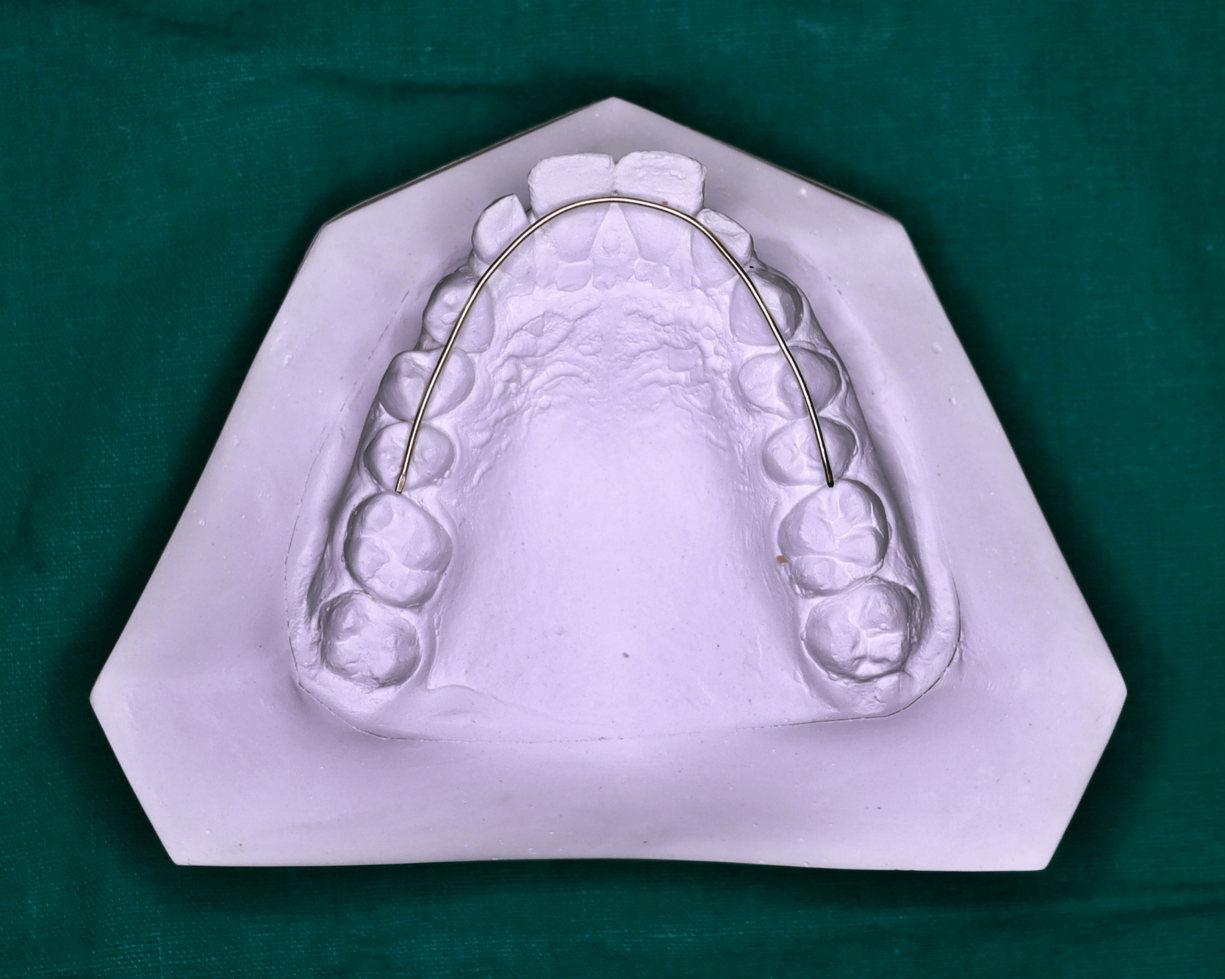
Measurement of arch length

The arch length was measured extending from the mesial surface of the first molar on one side, following the buccal cusps of the premolars and along the anterior teeth, continuing to the mesial surface of the first molar on the opposite side (Figure 2).

**Figure 3:**
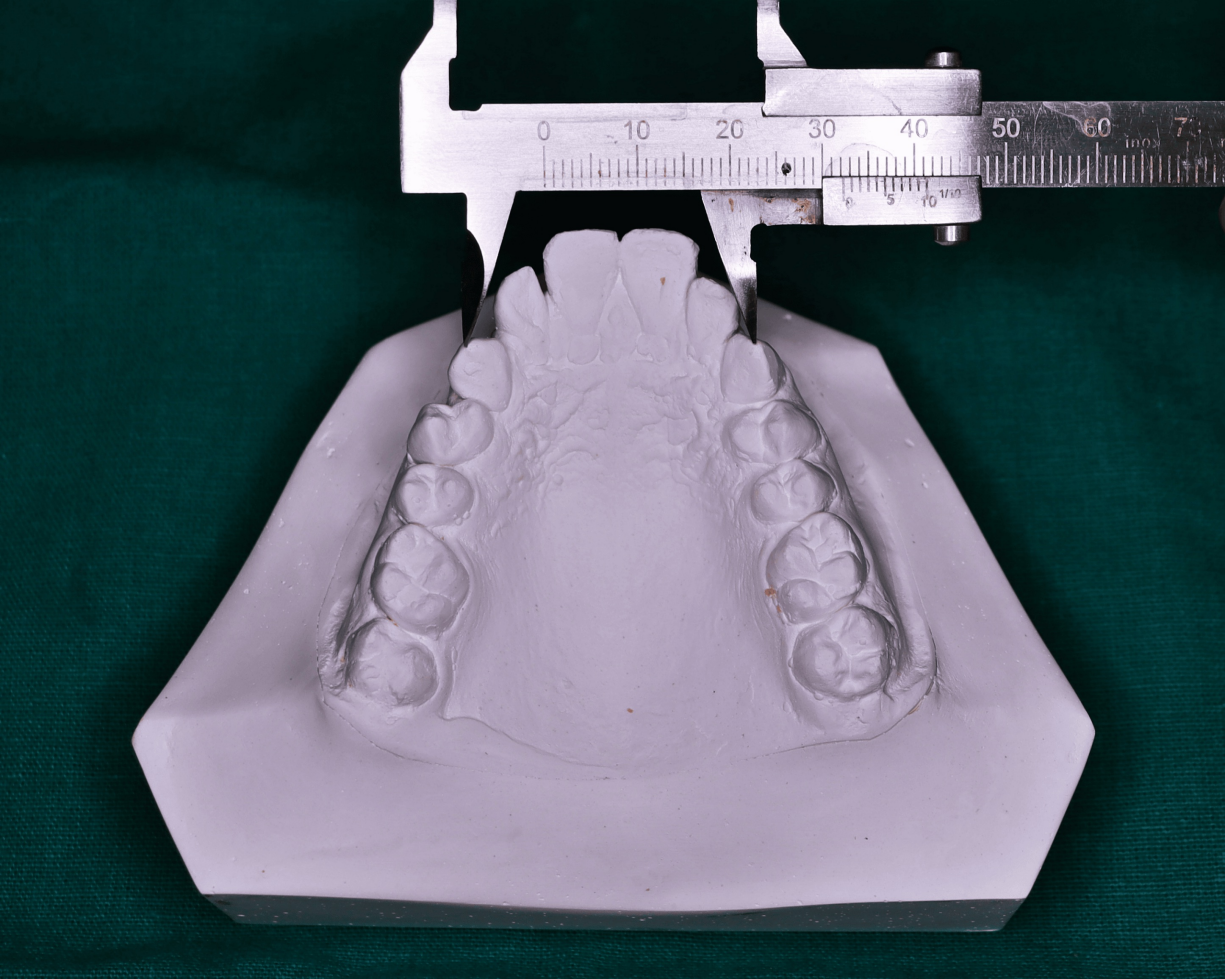
Measurement of intercanine width

Maxillary intercanine width was determined as the greatest linear distance between the cusp tips of the canines (Figure 3).

**Figure 4:**
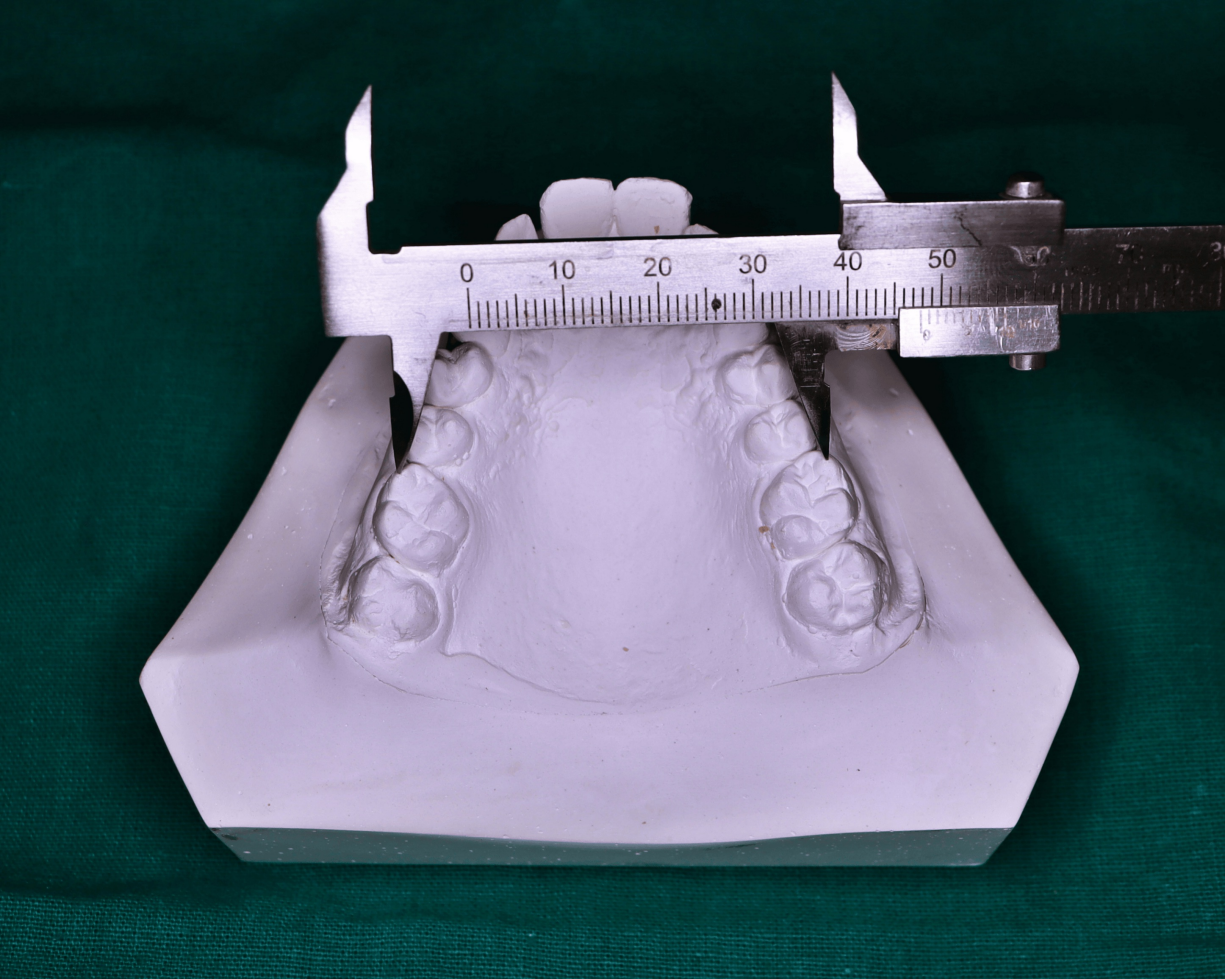
Measurement of intermolar width

Maxillary intermolar width was measured as the maximum linear distance extending between the mesiobuccal cusp tips of the molars, or the center of the wear facets on the cusp tips in cases of attrition (Figure 4).

**Figure 5:**
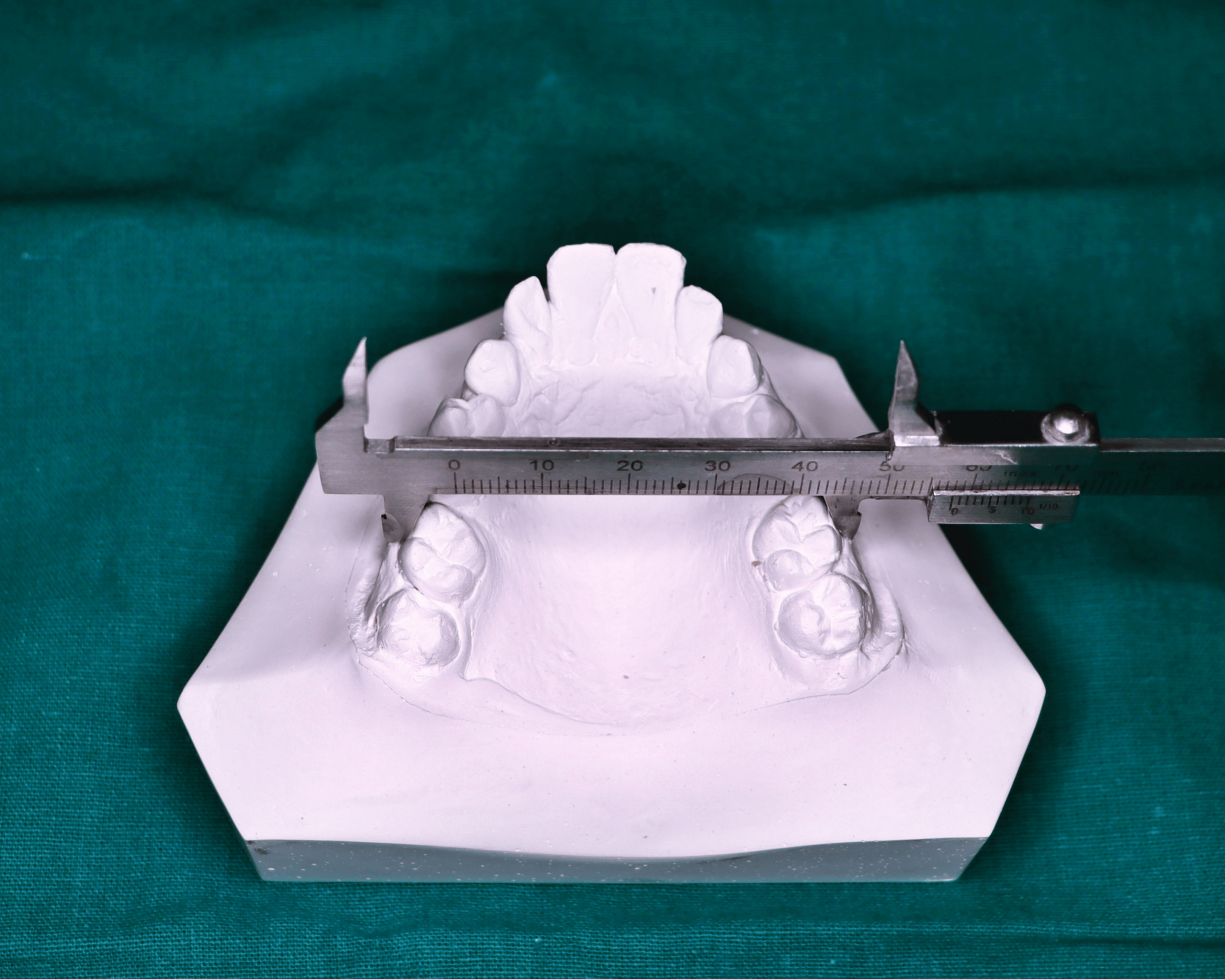
Measurement of interalveolar width

Maxillary interalveolar width was measured as the maximum linear distance at the level of the mucogingival junction above the buccal groove of the first molars (Figure 5) [17].

Landmarks on the dental cast were located and marked with a sharp 0.3 mm lead pencil. The measurements of the individual intercanine width, intermolar width, and interalveolar width of the maxillary cast were taken with Vernier calipers, and arch length with the help of a Brass wire, from the study models of each participant. The values obtained were tabulated and the means of the selected parameters were compared between both the groups to check for significant differences.

Statistical analysis

The analysis was performed with SPSS Statistics version 28 (IBM Corp., Armonk, NY). For continuous variables, descriptive statistics like mean and standard deviation (SD) were calculated. An independent samples t-test was used to determine the mean difference between two continuous variables, with statistical significance set at p<0.05. The intraobserver measurement error assessment, performed by the same examiner with a two-month interval, utilized Dahlberg's formula on 22 samples for each variable examined in the study-arch length, intercanine width, intermolar width, and interalveolar width for both groups. The results showed slightly lower errors in shallow palates (average: 0.34 mm) compared to deep palates (average: 0.42 mm). Despite this small difference, the overall error values were minimal, indicating a strong level of intraobserver reliability across both groups.

## Results

Table 1 provides the descriptive and comparative statistics of arch length, intercanine width, intermolar width, and interalveolar width for Groups A and B. It includes the mean values, standard deviations, minimum and maximum values, and the t-values and p-values to indicate statistical significance between the groups.

**Table 1:** Descriptive and comparative statistics of arch length, intercanine width, intermolar width, and interalveolar width between the groups *Statistical significance set at 0.05 SD: standard deviation

Variable	Group	N	Mean	SD	Min	Max	T	P-value
Arch length	A	44	71.82	5.56	61	80	-1.115	0.278
	B	44	74.17	4.52	69	82		
Intercanine width	A	44	33	2.09	29	35.5	1.261	0.221
	B	44	31.67	2.88	25	35		
Intermolar width	A	44	43.27	3.05	39	49.5	-5.246	0.001*
	B	44	48.71	1.81	46	51		
Interalveolar width	A	44	54.86	2.75	51	59	-2.601	0.017*
	B	44	57.88	2.8	54	62		

Comparative analysis using t-tests showed that there was no statistically significant difference in arch length between Group A and Group B (T = -1.115, p = 0.278). Similarly, no significant difference was found in intercanine width between the two groups (T = 1.261, p = 0.221). However, significant differences were noted in intermolar and interalveolar widths. Group B had a significantly greater intermolar width compared to Group A (T = -5.246, p = 0.001), and Group B also exhibited a significantly larger interalveolar width than Group A (T = -2.601, p = 0.017). These results highlight the specific dimensional differences between the two groups, particularly in the intermolar and interalveolar widths, where Group B demonstrated larger measurements compared to Group A.

The findings of this study suggest that in skeletal Class II patients with deep palates, there is a shorter arch length and significantly reduced intermolar and interalveolar widths, whereas the intercanine width remains unaffected.

## Discussion

Class II malocclusion is a frequently encountered orthodontic problem in various populations globally. Despite its widespread occurrence, effective management of Class II malocclusion has consistently posed a challenge for orthodontists in their routine practice. Skeletal and dental Class II malocclusion exhibit a range of clinical features and have been extensively studied by researchers [4,6]. Investigations have researched various aspects of this condition to aid in diagnosis and the exploration of diverse treatment approaches. This present study aimed to compare intra-arch dimensions of the upper arch, specifically arch length, intercanine width, intermolar width, and interalveolar width, in Class II Division 1 patients with different palatal depths.

Our findings revealed statistically significant differences in intermolar and interalveolar widths between groups with high and low palatal indices. Specifically, subjects with a high palatal index exhibited reduced widths. This aligns with the findings of Buschang et al. [8] who observed greater palatal height and a narrower maxillary dental arch in Class II Division 1 patients compared to Class II Division 2. Singh [9] also reported that Class II Division 1 subjects often have a deep palatal vault. On the contrary, Nahidh et al. [10] found lower palatal height in Class II Division 1 cases but did not observe significant differences compared to other malocclusions.

A study by Paoloni et al. [11] identified both deep and shallow palates in Class II Division 1 subjects, indicating a range of palatal morphologies within this malocclusion type. This variability could be attributed to individual growth patterns, genetic factors, or environmental influences such as oral habits. For instance, digit sucking is commonly associated with a deep palate, which in turn affects arch dimensions [18]. This hypothesis is supported by our findings, which suggest that Class II subjects with a high palatal index have lower intermolar and interalveolar widths compared to those with a low palatal index.

Our study further explored the relationship between palatal depth and intra-arch dimensions, revealing a strong correlation between them, in Class II Division 1 individuals. This intra-arch discrepancy, particularly a reduced maxillary inter-molar width, can contribute to functional aspects of distocclusion. This finding is in line with Varrela [12] who reported reduced maxillary widths in young children with Class II malocclusion, which tend to persist or worsen with age. Similarly, Tollaro et al. [19] attributed a narrower maxilla in Class II malocclusion to a shorter intermolar distance, emphasizing the role of transverse dimensions in malocclusion development. Rejman et al. [13] found that individuals with Class II Division 1 malocclusion typically have a narrower posterior dental arch width than those with normal occlusion, especially when crowding is present. However, Frohlich [14] reported no significant disparities in intra-arch widths between children with Class II malocclusion and those with normal occlusion, indicating that the relationship between arch dimensions and malocclusion may vary depending on the specific characteristics of the sample population.

Our study showed no significant correlation between arch length and intercanine width with palatal depth, diverging from some previous studies. This discrepancy might be due to differences in study design, sample characteristics, or measurement techniques. For instance, while the arch length and intercanine width are important parameters, they may be influenced by other factors such as dental eruption patterns, which were not controlled in our study.

The implications of our findings are significant for orthodontic practice. Recognizing the association between palatal depth and intra-arch dimensions in Class II Division 1 malocclusion can inform treatment planning [16]. Addressing transverse deficiencies through arch expansion protocols may mitigate the functional limitations posed by a deep palatal vault, thereby improving clinical outcomes. For example, early intervention with palatal expanders in growing patients can help correct transverse discrepancies and prevent the progression of malocclusion [16]. Furthermore, understanding the role of palatal depth in malocclusion can aid in identifying patients at risk for developing severe Class II discrepancies. Orthodontists can use this information to tailor treatment plans that address both the skeletal and dental components of malocclusion, ensuring comprehensive care [11]. For instance, patients with a deep palatal vault may benefit from treatments that expand the maxillary arch and promote mandibular growth, thereby improving the overall balance and harmony of the dental arches [16].

This study has a few limitations. For instance, the variance between the two genders between the groups was not taken into account. Additionally, the cross-sectional nature of the study limits our ability to draw causal inferences about the relationship between palatal depth and arch dimensions. Longitudinal studies tracking changes in palatal morphology and arch dimensions over time would provide more robust evidence on the development of Class II malocclusion and the effectiveness of different treatment approaches.

## Conclusions

Our findings reveal that subjects with Class II malocclusion, whether skeletal or dental, exhibit variations in palatal depth. Intermolar and interalveolar widths showed a correlation with palatal depth in Class II Division 1 individuals. Class II Division 1 patients with a deep palate displayed decreased intermolar and interalveolar widths compared to those with a shallow palate. This correlation offers important insights for orthodontic diagnosis and treatment planning and hence can aid in devising more personalized strategies for managing Class II malocclusion.
